# An Overview of Neonatal Lupus with Anti-Ro Characteristics

**DOI:** 10.3390/ijms22179281

**Published:** 2021-08-27

**Authors:** Malgorzata Gryka-Marton, Dariusz Szukiewicz, Justyna Teliga-Czajkowska, Marzena Olesinska

**Affiliations:** 1 Department of Biophysics, Physiology and Pathophysiology, Faculty of Health Sciences, Medical University of Warsaw, 02-004 Warsaw, Poland; dariusz.szukiewicz@wum.edu.pl; 2 Department of Systemic Connective Tissue Diseases, National Institute of Geriatrics, Rheumatology and Rehabilitation, 02-637 Warsaw, Poland; marzena.olesinska@vp.pl; 3 Department of Obstetrics and Gynecology Didactics, Faculty of Health Sciences, Medical University of Warsaw, 00-315 Warsaw, Poland; justyna.teliga@gmail.com

**Keywords:** autoantibodies, prenatal management, neonatal lupus

## Abstract

Neonatal lupus erythematosus (NLE) is a syndrome of clinical symptoms observed in neonates born to mothers with antibodies to soluble antigens of the cell nucleus. The main factors contributing to the pathogenesis of this disease are anti-Sjögren Syndrome A (anti-SS-A) antibodies, known as anti-Ro, and anti-Sjögren Syndrome B (anti-SS-B) antibodies, known as anti-La. Recent publications have also shown the significant role of anti-ribonucleoprotein antibodies (anti-RNP). Seropositive mothers may have a diagnosed rheumatic disease or they can be asymptomatic without diagnosis at the time of childbirth. These antibodies, after crossing the placenta, may trigger a cascade of inflammatory reactions. The symptoms of NLE can be divided into reversible symptoms, which concern skin, hematological, and hepatological changes, but 2% of children develop irreversible symptoms, which include disturbances of the cardiac stimulatory and conduction system. Preconceptive care and pharmacological prophylaxis of NLE in the case of mothers from the risk group are important, as well as the monitoring of the clinical condition of the mother and fetus throughout pregnancy and the neonatal period. The aim of this manuscript is to summarize the previous literature and current state of knowledge about neonatal lupus and to discuss the role of anti-Ro in the inflammatory process.

## 1. Introduction

Neonatal lupus erythematosus (NLE) is a system of clinical symptoms observed in children born to mothers with antibodies to soluble antigens of the cell nucleus. The main factors contributing to the pathogenesis of this disease are anti-Sjögren A Syndrome (anti-SS-A) antibodies, otherwise known as anti-Ro, and anti-Sjögren Syndrome B (anti-SSB) antibodies, otherwise known as anti-La. Recent publications have also shown the significant role of anti-ribonucleoprotein antibodies (anti-RNP) [1]. Seropositive mothers may have a diagnosed disease, e.g. Sjögren’s syndrome, systemic lupus erythematosus, rheumatoid arthritis, or mixed or undifferentiated connective tissue disease, or they can remain completely asymptomatic at the time of childbirth. Antibodies produced by the mother and found in her serum belong to the immunoglobulin G group and enter the fetal bloodstream from the time the placenta is formed, i.e., from the 12th week of pregnancy [2]. These antibodies may trigger a cascade of inflammatory reactions in the fetus and lead to the occurrence of neonatal lupus symptoms. The symptoms can be divided into reversible symptoms, which concern skin, hematological, and hepatological changes. They disappear spontaneously within a timeframe proportional to the decrease in the level of antibodies in the bloodstream of the fetus. A total of 2% of children develop irreversible symptoms, which include disturbances of the cardiac stimulatory and conduction system, i.e., a first- or second-degree atrioventricular block or even a complete cardiac block. Preconceptive care and pharmacological prophylaxis of a heart block in fetuses in the case of mothers from the risk group are important, as well as monitoring of the clinical condition of the mother and fetus throughout pregnancy and the neonatal period. The prevalence of neonatal lupus reaches 2% in mothers with anti-SS-A and/or anti-SS-B antibodies. The first scientific reports on children with neonatal lupus symptoms date back to 1954, but many questions remain unanswered, although more than 60 years have passed since the discovery of the disease. The aim of this manuscript is to summarize the current state of knowledge about neonatal lupus, including its pathogenesis, the clinical picture of mothers and their children, prophylactic actions in the prenatal period, and therapeutic actions directed towards neonates and infants.

## 2. Previous and Current Diagnostic Methods 

‘LE’ cells (lupus erythematosus) play a major role in the pathogenesis of lupus. They were discovered by Hargraves et al., who described them as ‘mature multinuclear granulocytes that phagocytosed Feulgen-colored nuclear material’ [3]. The process begins with the interaction of antibodies contained in the serum and cell nuclei. The nuclei undergo homogenization and swell. After that, they are released from the cells as ‘LE corpuscles’. In the presence of a complement, the destroyed nuclei are phagocytized, which results in the formation of LE cells [4]. Testing positive for LE cells was the first of the ‘immunological disorders’ among the classification criteria for systemic lupus erythematosus (SLE) and has been recognized as a criterion for more than 30 years [5]. This was omitted in the modified criteria introduced by the American College of Rheumatology (ACR) in 1997 [6]. Currently, the gold standard in the diagnostics of systemic connective tissue diseases is anti-nuclear antibodies (ANA) assessed by indirect immunofluorescence (IIF). Specificity is assessed by enzyme-linked immunosorbent assays (ELISA) or immunoblots. Comparing historical and current methods, a conclusion can be drawn that LE is caused by double-stranded native DNA antibodies (anti-dsDNA) [4]. At present, LE cells can be assessed in some laboratories, but their diagnostic value in terms of modern techniques has significantly decreased.

### 2.1. Historical Beginnings of Neonatal Lupus

Studies on neonatal lupus date back to the 1950s. In 1954, McCuistion and Schoch [7] were the first to mention skin lesions in a child whose mother was diagnosed with systemic lupus erythematosus. However, it was Hogg in 1957 who first used the term ‘neonatal lupus’ when describing a case of a male neonate with congenital severe lupus erythematosus. The mother had been diagnosed 30 months prior to labor with subacute cutaneous lupus erythematosus. The child was delivered in the 38th week of pregnancy. The physical examination revealed a total atrioventricular block with an atrial rhythm frequency of 150 beats/min, whereas the frequency of the ventricular rhythm oscillated between 38–48/min. The diagnosis was based on the results of the autopsy and diagnosed endocardial fibroelastosis [8]. In subsequent studies from 1957 to 1966, a correlation between cutaneous and cardiological symptoms in children was confirmed, and the hypothesis about lupus factor transplacental transport was maintained [9,10,11].

### 2.2. Pathogenesis

It is difficult to discuss neonatal lupus without mentioning its pathogenesis because it is the source of new diagnostic and therapeutic opportunities. It is currently known that the onset of neonatal lupus is related to anti-nucleus antibodies in the mother. These antibodies are anti-nuclei antibodies, belonging to the group of autoantibodies against extractable nuclear antigens. This group of antibodies also comprises antibodies against the following antigens: Sm, RNP, SS-A/Ro, SS-B/La, Scl-70, Jo-1, Pm/Scl, Mi-1, Mi-2; however, these are anti-SSA/Ro, anti-SSB/La, and anti-RNP antibodies, which play a key role in neonatal lupus pathogenesis. Antigens for these antibodies are located intracellularly in the cytoplasm or cell nucleus. They are expressed on the cell surface only if the cell is to be apoptosis assisted.

### 2.3. Anti-SS-A/Ro Antibodies 

Anti-SS-A antibodies, also referred to as anti-Sjögren Syndrome A, were first described in 1970 in a patient with Sjögren’s syndrome. They are otherwise called anti-Ro, after the name of the patient (Rose) in which it was first detected. They are directed against four different antigens, each of which consists of a complex of low molecular weight RNA (micro-RNA) and protein, whose molecular weight may be 45, 52, 54, or 60 kDa. Only targeted antibodies against molecule SS-A/Ro52 and SS-A/Ro60 are used in everyday medical practice, although older studies do not distinguish between Ro52 and Ro60 antibodies, defining them together as anti-SS-A/Ro [12]. Ro60 was found to be an RNA binding protein [13], while the second Ro52 protein was ubiquitin E3 ligase, targeting cytosolic virus–antibody complexes [14]. It remains unclear why antibodies to these two functionally unrelated proteins are often detected together in the serum of patients [15]. Antibodies against SS-A/Ro52 are also referred to as SS-A/Ro52/Tripartite Motif Protein 21 (TRIM21). The nomenclature of anti-SS-A antibodies is a source of inconvenience in scientific manuscripts. Some authors, e.g., Robbins et al. suggested changing the nomenclature of antibodies against soluble cellular nucleus antigens (ENA) to avoid misunderstandings by abandoning the anti-SS-A label in favor of anti-Ro52/TRIM21 or anti-Ro60 antibodies for more precise expression. They based their suggestion on a paper published in 2019 that assesses the clinical usefulness of anti-Ro52 and anti-Ro60 [16]. Additionally, in their study evaluating the serum of 13,032 patients for anti-SS-A antibodies, they demonstrated that in the group of anti-Ro60 positive (anti-Ro52 negative) patients, the prevalent diagnosis was systemic lupus erythematosus, whereas in the group of patients with antibodies against both Ro52 and Ro60 antigens, the most common diagnosis was Sjögren’s syndrome. The dominant group consisted of patients with isolated anti-Ro52 antibodies who had a wide range of diagnosed diseases, including cancer and infections, but in terms of the autoimmune diseases, the most frequent diagnoses were muscle inflammation and systemic sclerosis. Mung and Thomas obtained the same results in their studies [17]. The table below illustrates the distinction between the two antibodies (Table 1).

### 2.4. Anti-Ro52

The Ro52 antigen belongs to the TRIM21 family of receptors and a family of E3 ubiquitin ligases; it takes part in protein ubiquitination and triggers pro-inflammatory action (by affecting interleukin 2 synthesis) and is also involved in cell apoptosis [18,19,20,21]. TRIM21 is a receptor present in the cytoplasm as well as in proinflammatory environments in the cell nucleus. It binds to the Fc region of immunoglobulin G [22]. After being stimulated by interferon and binding to TLR (Toll-like receptors), the TRIM21 receptor also interacts with transcription factors for interferon regulatory factor (IRF). The reported substrates for Ro52 replication include IRF3, IRF5, IRF7, and IRF8, and through these transcription factors, they regulate interferon type 1 levels and cytokine production [18]. Additionally, Ro52/TRIM21 can also regulate the activation or proliferation of T cells, and the overexpression of this receptor can increase the synthesis of IL-2 [23]. Patients with lupus erythematosus are diagnosed as having anti-Ro52 antibodies. Anti-Ro52 antibodies show a strong correlation with hypersensitivity to light (they are present in one third of patients with the cutaneous type of systemic lupus erythematosus). Other studies conducted by Yoshimi have shown the relation of anti-Ro52 antibodies with late-onset systemic lupus erythematosus (average age of 50 years), hypersensitivity to UV radiation, and dermatitis and hematological disorders (pancytopenia or deficiency of one of the hematopoietic system classes) [24]. The most clinically significant manifestation of positive results for anti-Ro52 antibodies is congenital heart block (CHB). Salomonsson et al. determined the serological profile of mothers of infants with CHB, showing that 95% of them had a positive anti-Ro52 antibody titer, and the frequency of Ro60 and La antibodies was 63% and 58%, respectively [25].

In addition, they occur in patients with Sjögren’s syndrome and systemic scleroderma. In case of autoimmune myositis, the presence of anti-Ro52 often coincides with other autoantibodies, but it may be the only positive specific antibody [26,27]. Anti-Ro52 also occur in patients with interstitial lung disease [28], or autoimmune liver diseases such as primary biliary cirrhosis (PBC) and autoimmune hepatitis 1 [29]. Anti-Ro52 antibodies are diagnostically significant because they coincide with other more disease-specific antibodies. Alone, they are not specific due to their common occurrence in other non-autoimmune diseases. Their higher titer is associated with proliferative diseases and infections [16]. The most sensitive and specific technique for their detection is the ELISA method, but they can also be detected with the indirect immunofluorescence (IIF) method or by using human cancer cells transfused with SS-A/Ro antigen as a substrate [30]. 

### 2.5. Anti-Ro52 with Epitope p200 

Anti-Ro52 epitope p200 (anti-amino acid antibodies in positions 200–239 of the Ro52 antigen) is the most likely to cause a complete heart block in the fetus and was found to be the highest risk factor for heart damage. Standberg has shown in his studies that mothers of children with a second- or third-degree atrioventricular block had significantly higher p200 antibodies levels than mothers with rheumatic disease, whose children had heart rates within the normal range. A significant difference was observed in anti-p200 levels between the groups with the first-degree block and those with the second- and third-degree block, compared to fetuses with normal conduction. The use of p200 antibodies as the second stage of analysis during pregnancy in the case of a mother with anti-Ro52 antibodies increased the positive predictive value for fetal heart block (AVB type I, II, or III) from 0.39 (0.27–0.51) to 0.53 (0.37–0.68) [31]. In summary, antibodies against Ro52p200 may occur in women with healthy children, but their levels are significantly higher in mothers of children with congenital heart block and are suggested to be a significant marker in the assessment of the risk of developing an atrioventricular block.2.6. 

### 2.6. Anti-Ro60

Ro60 is a protein with a molecular weight of 60 kD. It is also called TROVE2. The Ro60 protein consists of two domains: one of them is similar in structure to the von Willebrand factor. It is also present in extracellular space and takes part in cellular adhesion. The second domain of the alpha helix structure resembles a cylinder shape and is responsible for binding nucleotide acids [32]. The Ro60 antigen is a protein in the hY-RNA complex, which may bind abnormally corrugated non-coding RNA, contributing to its degradation [32]. Research has shown that mammalian cells and bacteria devoid of the Ro60 protein are hypersensitive to ultraviolet radiation; hence, Ro60’s function may be important when exposed to environmental factors [15]. Chen proved in 2003 that the Ro60 protein increases cellular survival after exposure to UV radiation [33], and this thesis was further demonstrated by other researchers in assessing that Ro60 accumulates in the cell nucleus after UV radiation or oxidative stress [33,34]. The anti-Ro60 antibody appearing in the specification as the only specific antibody (monopositive) shows a strong correlation to systemic lupus erythematosus, and its association with coexisting antiphospholipid antibodies, especially with anticardiolipin antibodies and lupus anticoagulant, cannot be excluded [16]. The presence of autoantibodies in the serum precedes clinical symptoms by several years; the earliest detectable autoantibodies are against Ro60 [35].

### 2.7. Which Factor Triggers the Production of Anti-Ro60 Antibodies?

Initially, it was believed that the first antibodies in systemic lupus patients were formed by the molecular mimicry mechanism between the Epstein–Barr virus nuclear antigen-1 (EBNA1) and the Ro60 protein. However, no similarity has been found in the amino acid sequences of EBNA1 and Ro60; thus far, no explanation has been given as to why EBNA1 mimics the Ro60 protein [36]. Subsequent studies have suggested that molecular mimicry between the Coxsackie virus protein and Ro60 is the cause of anti-Ro60 antibody production in patients with Sjögren’s syndrome in the case of infection with this virus [37]. A recent study has led to a new hypothesis that the proteins of some commensal bacteria may contain epitopes imitating human Ro60 parts and thus act as initiators of the autoimmune anti-Ro60 response. For example, a peptide from the domain of von Willebrand factor type A (vWFA) associated with the oral cavity micro-organism Capnocytphaga ochracea was the strongest activator of T cells reactive to Ro60. Another factor stimulating their activity was Escherichia coli bacteria expressing vWFA [38]. These results indicate that peptides of commensals in a healthy human can activate T cells reactive to Ro60 [38]. In 2018, Greiling published a study in which he identified orthologs of Ro60 in a subgroup of human commensals bacteria colonizing the skin, mouth, and intestines and confirmed the presence of these orthologs in patients with lupus and in a control group of healthy individuals. Some Corynebacterium, Propionibacterium, and Bacteroides species have been shown to code Ro60 orthologs with high sequence similarity to human Ro60 [39].

### 2.8. Anti-SS-B/La Antibodies

Anti-SS-B/La (anti-Lane antibodies) were named after a patient named Lane in whom these antibodies were found for the first time. They are directed against phosphoprotein with a molecular weight of 48 kDa, which is associated with RNA polymerase III [40]. They are present in patients with Sjögren’s syndrome as well as in patients with systemic lupus erythematosus (10–20%). In Sjögren’s syndrome, antibodies against SS-B/La are almost always present together with antibodies against SS-A/Ro [41]. The presence of anti-La antibodies in the absence of anti-Ro antibodies is very uncommon, and cases of CHB associated with only anti-La antibody positivity make up less than 1% of the incidence of autoimmune CHB in the literature [42].

### 2.9. Anti-RNP Antibodies

Anti-ribonucleoprotein antibodies (RNP) are directed against three antigens. For this reason, some authors specify three types of antibodies. The RNP antigen belongs to a group of small nuclear ribonucleoproteins (snRNP) containing low molecular RNA with high uridine content (U-RNA) and various core proteins with a molecular weight of 70 kDa (U1), 33 kDa (protein A), and 22 kDa (protein C). The RNA components are labelled as U1 to U6. The RNP antibody is heterogeneous and directed against various epitopes, the most important of which has a molecular weight of 70 kDa (U1). Anti-U1-snRNP antibodies are detected in high titers in 95 to 100% of patients with mixed connective tissue disease (MCTD). The presence of anti-U1-RNP antibodies in the serum is required by all MCTD diagnostic criteria. These antibodies, found in all patients and often in high titers, are not always present from the beginning of the disease. Their titers may fluctuate and persist during the disease activity, often also during clinical remissions, but their titers frequently decrease or disappear during periods of improvement. They may also be present in patients with systemic lupus erythematosus, rheumatoid arthritis, or Sjögren’s syndrome, but in these patients, their titer does not correlate with the activity of the disease [41]. Antibodies against nuclear ribonucleoprotein (nRNP) detected by indirect immunofluorescence (antigen-tissue substrates and HEp 2 cells) give a speckled (granular) type of light. For more precise identification of individual autoantibodies, the immunoenzymatic method (ELISA) and Western-blotting technique are used, which allows one to obtain additional data, e.g., the molecular weight of antigens and the subfraction composition of individual antigens [30].

### 2.10. Transplacental Transport of Antibodies

The onset of neonatal lupus is related to the antibodies received from the mother. ENA antibodies belong to the immunoglobulin G group, within which four subclasses are differentiated: IgG1, IgG2, IgG3, and IgG4. IgG is transported by transcytosis via neonatal receptors for Fc IgG fragments present on placental trophoblast cells. The neonatal Fc receptor (FcRn) is a transbone heterodimeric glycoprotein with a major histocompatibility complex (MHC)-like structure, but unlike MHC class I, FcRn is unable to bind peptides (antigens); however, it interacts with Fc IgG and albumin. IgG and albumin are the main plasma proteins accounting for about 80% of the total plasma protein pool and have the longest half-life (about 20 days). This suggests that they can be protected against catabolism by an inverse relationship between the serum concentration of both of these proteins and their half-life. Their catabolism is directly proportional to their blood levels and increases with hyperimmunity. IgG catabolism is fully dependent on FcRn. The neonatal receptor functions throughout life, maintaining IgG and albumin levels by protecting them from intracellular degradation. This property of the FcRn receptor is used to prolong the half-life of biological drugs such as etanercept [43]. The presence of an Fc fragment of human immunoglobulin gives the drug a longer half-life. FcRn–IgG interaction, as well as FcRn–albumin interaction, both take place in an acidic environment (optimally at pH 5.0–6.5) and not in a physiological environment. Brambell was the first to present a hypothesis about the transport of IgG through the placenta, on the basis of which the data were supplemented with current reports [44,45]. The mechanism of transporting maternal IgG class antibodies through the human placenta syncytiotrophoblast begins with the absorption of IgG into the cell by liquid phase endocytosis and leads to early/sorted endosomes; then, in the acidic endosomal environment, IgG molecules are bound by FcRn and are thus protected against degradation by lysosomal enzymes. FcRn–IgG complexes in transcytotic bubbles are then transferred to within the chorionic villous stroma, while IgG molecules that have not bound to FcRn are directed to the path of degradation (by multi-bubble late endosomes to lysosomes). FcRn releases IgG within the chorionic villous stroma (pH 7.4). After the dissociation of IgG, the FcRn receptor returns to the apical syncythiotrophoblast surface in transcytic vesicles. As a result of this mechanism, fetal serum antibodies can reach slightly higher concentrations than those of the mother serum, as is the case with the IgG1 subclass, while for the IgG2 subclass, fetal serum antibody concentration can be even lower, while the level of the IgG3 and IgG4 subclasses in the serum of the mother and fetus are equal [46]. Tseng investigated the matter regarding which subclasses the placental antibodies in mothers with children with congenital heart block belong to. The study group consisted of 32 patients with anti-SS-A and/or anti-SS-B antibodies, 23 of whom had children with congenital atrioventricular block, and 11 of whom had healthy children. It has been shown that IgG1 and IgG3 were the main subclasses represented by anti-SS-B and anti-Ro52. All subclasses, including IgG2 and IgG4, were observed in approximately one third of the anti-Ro52 and anti-SS-B responses. However, anti-Ro60 antibodies were limited to IgG1, with a few exceptions. No significant differences were observed between affected and unaffected pregnancies in terms of the mother–fetus ratio of any of the antibodies subclassesIn summary, there were no significant differences in subclass profiles between mothers who gave birth to children with cardiac rhythm disorders and those whose offspring were healthy [47]. 

### 2.11. What Happens in the Heart of a Fetus When the Antibodies Pass the Barrier?

Maternal sera containing anti-Ro antibodies may bind cross-reactive epitopes on calcium-regulating molecules such as ion channels type L and T [48], which leads to the dysregulation of calcium homeostasis, calcium overload, and cellular apoptosis. Anti-Ro60 and anti-La autoantibodies may then bind their cognate antigen on the surface of apoptotic cells and start the inflammation pathway of removing cardiac cells. This stage probably corresponds to clinical fetal AV time prolongation or AV block stage I [49]. Depending on the presence of fetal susceptibility genes such as HLA, the local inflammation may either be resolved, leading to normalization of fetal AV conduction, or be propagated and amplified, leading to fibrosis and calcification and the development of permanent fetal AV block III. Analyses of single-nucleotide polymorphism genotyping in 86 families reveal that HLA-DRB1*04 and HLA-Cw*05 alleles are associated with a higher risk of CHB, while DRB1*13 and Cw*06 appeared to be protective factors [50]. It remain unknown which factors lead to progression the conduction failure. [51]. Due to the inflammation process starting from anti-Ro transplacental transport and binding apoptotic cardiomyocytes, the following processes take place: deposition of C4 complement, calcification, and fibrosis [52,53]. Recent data show that the maternal anti-Ro antibody induced an inadequate pathway to remove the apoptotic cardiac cells. The non-inflammatory pathway is replaced by engulfment by inflammatory process by macrophage opsonization, leading to macrophage activation and the production of pro-inflammatory and pro-fibrotic cytokines such as TNFα and TGFβ [54,55].

### 2.12. Pathogenetic Role of Macrophages and Interferon Type I

There is special type of macrophages expres high levels of sialic acid-binding Ig-like lecithin 1 (SIGLEC-1) on the cell surface. Siglec-1-positive macrophages were found in the cardiac lesions in fetus with CHB [56]. These cells can increase the inflammatory response by the enrolment of other mononuclear cells. Since the expression of SIGLEC-1 is upregulated by type I interferon (IFN), recently, the role of IFN and IFN-stimulated genes in the pathogenesis of CHB have been investigated. Type I IFN upregulates Ro52 and stimulates apoptosis, so many studies have concentrated on finding out whether there is a correlation with CHB [57]. Lisney et al. demonstrated the correlation that mothers of children with CHB had a significantly higher expression of SIGLEC-1 and IFN-α in comparison to mothers with unaffected children [58]. Hedlung et al. observed an increased expression of IFN-regulated genes and elevated plasma IFNα levels in anti-Ro/La-positive women and in their newborns [59]. In addition, a correlation between IFN scores in mothers and their offspring has been identified, whereby the evaluation of the maternal IFN score could be used as novel biomarker for CHB risk [59].

### 2.13. Epidemiology 

The incidence rate of NLE is about 1 in 12,500 to 20,000 live births and is slightly higher in female and premature babies [60]. However, the true prevalence of NLE has not been established due to the high proportion of unrecognized cases. Approximately 40–60% of mothers remain asymptomatic, when NLE has already been diagnosed in neonates [61].

### 2.14. Risk Factors of NLE

It has not yet been explained why some fetuses suffer from congenital heart block and some do not, despite the same developmental conditions. However, we started to differentiate some conditions that predispose the occurrence of neonatal lupus erythematosus. Jill Buyon has played a significant role in exploring the issue of neonatal lupus by publishing many works on this matter. Buyon was the first to notice the correlation between the specificity and number of mother’s antibodies crossing the placenta barrier and the risk of developing the congenital heart block in a fetus. The study published by her in 1993 proved an increased risk of fetal heart complications when anti-SS-A and anti-SS-B antibodies were found in mothers, especially when specific anti-Ro52 antibodies were detected. Buyon also showed the lack of correlation between the type of anti-SS-A or anti-SS-B antibodies and clinical symptoms of neonatal lupus. In the same study, Buyon showed that, in women with low antibody titers, the risk of newborn lupus was low [62]. Jaeggi has unequivocally confirmed this thesis. In his study, he observed mothers with low antibody titers. It was determined that none of their fetuses had cardiological complications in the form of heart block [63]. 

Apart from this, there are studies being conducted currently in order to find fetal factors associated with susceptibility to the development of total heart block. As we know today, neither gender nor tissue compatibility antigens (MHC) influence susceptibility, although some researchers have shown slightly higher morbidity in female fetuses [60]. It has been shown that risk factors for CHB development may be related to the polymorphism of TGF beta cytokine (it has profibrotic effect [64]) as well as the presence of maternal microchimerism [65]. Rivera et al. carried out a genotype analysis for TNFα, TGFβ, and interleukin 1α polymorphisms in patients whose children were born with newborn lupus. He showed a higher incidence of the TGFβ TT genotype in mothers who developed lupus erythematosus than in those who remained asymptomatic. TGFβ reaches lower concentrations when no TT genotype is detected; perhaps the lack of the TT genotype may have protective functions on asymptomatic mothers against disease development. Reports of polymorphisms for TNFα (−308A/G) and IL1α (−889C/T) in SLE led Rivera to study these cytokines [66]. While the TNFα-308 A and IL1α-889 C/C allele was found to be significantly related to SLE in comparison to the control group, none of them were significantly higher in SLE compared to asymptomatic mothers. In summary, it is possible that further studies will prove to be valuable in the study of TGFβ polymorphisms to determine the risk of autoimmune disease progression.

Ambrosi et al. conducted studies on a Swedish patient population and studied the effects of fetal sex, number of children born by the mother, and the mother’s age and birth time on the occurrence of congenital heart block. Neither the fetal sex nor the number of children born by the mother were correlated with the occurrence of heart block. Pregnancies which led to the birth of a child with heart block correlated with a higher age of the mother, the duration of the second trimester of pregnancy in winter months, childbirth in summer, and lower levels of vitamin D3 [67].

### 2.15. Characteristics of the Mothers 

The mothers of children with neonatal lupus erythematosus are characterized by the presence of anti-SS-A and/or anti-SS-B antibodies, but in some cases, anti-RNP antibodies are also found, even without the presence of anti-SS-A antibodies. Despite their seropositivity, the mother does not always have a diagnosed systemic connective tissue disease. Many authors have made a prospective evaluation of the clinical characteristics of patients whose children suffer from neonatal lupus. 

Some mothers are asymptomatic when they give birth, and their serological status is determined only after the diagnosis of abnormalities in the child. Although the women have no symptoms at the time of procreation, this does not protect them against development of the disease. Rivera et al. assessed 321 mothers listed in the Registry for Neonatal Lupus kept by American scientists. The patients were observed for 6 months to a few years. Half of the women with no visible symptoms developed the autoimmune disease. Out of 26 women who developed the disease, 14 were diagnosed with undifferentiated connective tissue disease, 7 with Sjögren’s syndrome, 4 with systemic lupus erythematosus and 1 with Sjögren’s syndrome [66]. The probability of developing lupus erythematosus within 10 years after giving birth to a child with NLE was calculated at 18.6%, and the probability of developing Sjögren’s syndrome in such a patient was 27.9%. As was reported by Lawrence, mothers who gave birth to children with skin manifestations are slightly more likely to develop an autoimmune disease than those who gave birth to a child with heart block [68], but in the Rivera study, such a correlation was not found [66]. Patients who tested positive for both anti-SS-A and anti-SS-B antibodies were almost twice as likely to develop the disease as patients with only anti-SS-A antibodies [66].

Zuppa et al. evaluated 50 patients who tested positive for anti-SS-A/Ro antibodies and monitored their children for 9 months. The most frequent maternal autoimmune disease accompanying symptomatic neonatal lupus was systemic lupus erythematosus (25 patients). A total of 15 mothers had primary Sjögren’s syndrome, and 5 mothers were diagnosed with systemic lupus erythematosus and Sjögren’s syndrome; 2 patients had mixed connective tissue disease, and 3 patients were asymptomatic without diagnosis of any systemic connective tissue disease [69]. The results of the other studies are presented in Table 2. 

In summary, the above data indicate the need to monitor asymptomatic mothers in terms of the potential development of systemic autoimmune diseases and to ensure the in-depth monitoring of the condition of the fetus and mother with diagnosed SLE or arthritis to protect their well-being.

### 2.16. Characteristics of Neonatal Lupus

Neonatal lupus is a rare disease and an example of passively acquired autoimmunization. We can define it as a set of clinical signs observed in the children of mothers with anti-SS-A and/or anti-SS-B antibodies [42]. The clinical picture of the disease is multiform; the symptoms can be temporary (for example skin lesions) or include organ changes such as hematological, hepatological, or irreversible disorders, such as congenital heart block. Non-cardiological symptoms are more frequent than cardiological ones and disappear in the first months of life together with the disappearance of the mother’s antibodies in their serum. The incidence of symptoms has been discussed in many studies, e.g., in that of Zuppa, Cimaz, Motta, Boros [69,71,75,76]. These data are summarized in Table 3. Cimaz et al. assessed the incidence of different symptoms in children, whose analysis gave the following results: 16% for skin lesions, 27% for hematological complications, and 26% for increased activity of liver enzymes [71]. According to the data obtained by Cimaz, cardiological complications were observed in 1.6% of children born as the first child by their mothers who tested positive for anti-SS-A antibodies [71]; according to Brucato’s studies, the number is 2% [70]. Izmirly examined 77 children whose elder siblings had the cutaneous form of neonatal lupus. He estimated the risk of the general recurrence of any manifestation of neonatal lupus in the second pregnancy at 49%, the recurrence of the cutaneous form at 30%, the cardiological form at 18%, and the hematological form at 1% in children [77]. 

Sato et al. published an interesting case of a female neonate born in the 36th week of pregnancy by caesarean section because her mother went into labor. The mother was 32 at the time of delivery and did not show any symptoms of connective tissue disease. However, 3 years before, when she was 29, she was monitored for symptoms of lupus erythematosus due to erythema on her face. At that time, she did not meet the classification criteria and was not diagnosed as suffering from the condition. After birth, the child was observed to have the following conditions: skin lesions in the form of a ring-shaped erythema and subcutaneous tissue atrophy on the face, trunk, and back. In addition, fetal growth restriction (FGR) was diagnosed. Subsequent studies showed a slight increase in hepatic aminotransferases activity, thrombocytopenia, and mild heart failure with no atrioventricular block. Serologically, the neonate showed elevated moderate titers of anti-SS-A antibodies and high titers of anti-SSB. Anti-DNA and anti-RNP antibodies were absent, and complement components were not lowered. The mother’s serological test results were the same as those of the child. In order to broaden the diagnosis of inflammatory diseases of systemic connective tissue diseases, a histological examination of the minor salivary gland was performed in the mother, which showed lymphocytic infiltration. The mother was diagnosed with Sjögren’s syndrome. All symptoms in the neonate disappeared at the age of 7 months, without any health complications. 

The researchers carried out a histopathological examination of the placenta, which revealed a collapse of capillaries in the placenta but no visible thrombus formation; massive infiltration of inflammatory cells and apoptotic cells with hematoxylin and eosin (HE) staining was found. C4d deposition was visible in the endothelial cells of the terminal villi of placenta and umbilical cord vessels. However, no C4d deposition was detected in the maternal vessels. The findings of the researchers suggest that maternal antibody reactions in the placenta induce fetal organ damage but do not attack the placenta itself or the maternal vessels. A hypothesis was posed that the complement is activated by maternal antibodies in the fetal vessels through the classical pathway, and, later on, inactive C4d binds to the surface of the fetal endothelium. Covalently bound C4d anchors tightly in the tissue, which is useful as a trace of tissue damage through antibodies. Although it is an inactive product of the complement cascade division, it is postulated that C4d may have the function of a new marker of tissue destruction by autoimmune phenomena [78]. Detection of deposited C4d complexes is considered nowadays to be the gold standard for the diagnosis of acute humoral resection of kidney transplantation [79]. However, further research is needed to demonstrate the usefulness of C4d deposition as a new NLE marker [80]. 

It is worth mentioning that C4d deposition in the placenta also occurs in the majority of mothers with systemic lupus erythematosus, antiphospholipid syndrome, and pregnancy-induced arterial hypertension. At the same time, it is associated with complications for the fetus, namely, preterm birth and fetal growth restriction (FGR) [81]. It is suggested that C4d could be used as a biomarker used to evaluate the future risk of FGR and to control the disease during pregnancy in the case of these patients [81]. In summary, Sato et al. determined the diagnosis of the newborn on the basis of skin symptoms, antibody levels in the serum of the newborn and mother, and histopathological examinations of the placenta [80].

### 2.17. Skin Lesions

**Figure 1:**
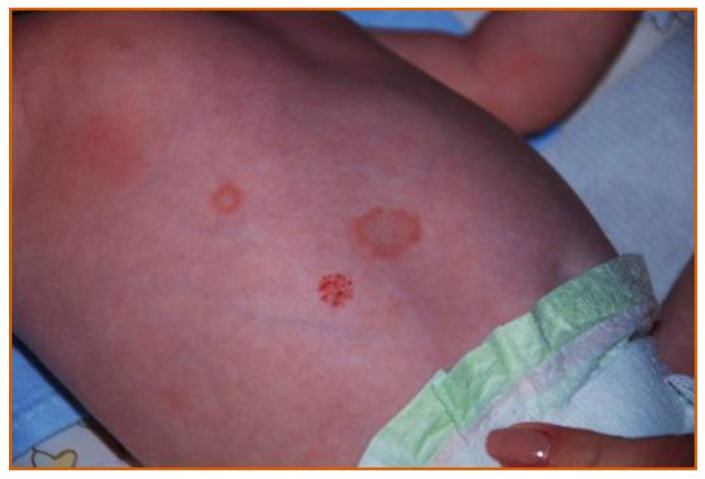
Skin lesion in a one-week-old neonate with NLE.

Neonatal skin lupus occurs in 5−16% of infants exposed to anti-Ro and/or anti-La antibodies. Exposure to anti-SS-B antibody and female sex are risk factors for developing skin lesions [82]. Dermatological lesions may be present at birth but also often appear during the first few weeks of life. Ring-shaped erythematous or polycystic plaque with or without scars characterizes NLE and appears mainly on the scalp, neck, or face. Periorbital erythema, referred to as ‘the eye of the raccoon’ or ‘the eye of the owl’, is a common feature. The prognosis for skin lesions is usually good. UV light can be trigger the development of lesions or can exacerbate existing ones [83]. Histologically, the lesions are similar to those of subacute lupus with hyperkeratosis, epidermal atrophy, basal degeneration, intracellular oedema, and perivascular infiltrations by inflammatory cells. In addition, direct immunofluorescence reveals complement and immunoglobulin complexes. Usually, skin lesions do not require therapeutic intervention, are self-limiting, and disappear with the disappearance of maternal antibodies in the fetal serum within a few months after birth. The enclosed photograph (Figure 1) depicts a neonate with skin lesions characteristic for neonatal lupus. The child was a patient at the Duchess Anna Mazowiecka Clinical Hospital in Warsaw. 

### 2.18. Cardiological Complications

Cardiological complications in neonatal lupus may include transient arrhythmias; first-, second- or third-degree atrioventricular block; dilated cardiomyopathy; and fibroelastosis of the endocardium. Atrioventricular block is the most common cardiological complication of neonatal lupus; heart fibroelastosis is much less common.

### 2.19. Congenital Heart Block as the Most Serious Complication

The population prevalence of atrioventricular block is estimated at 0.005% [84], i.e., 1 in every 15,000–20,000 births. The risk of heart block in the population of anti-SS-A-positive mothers is higher and equals 1–2% in the first pregnancy or when children from previous pregnancies did not suffer from neonatal lupus [70,76]. If elder siblings had neonate lupus, the risk of disease for the subsequent child increases to 10–20% [85,86]. Li et al. suggest that congenital heart block frequency was significantly lower in Asians than in Caucasians [73]. Atrioventricular block is most often prenatally diagnosed between 18 and 24 weeks of pregnancy, which is also the time when a woman undergoes a mandatory fetal ultrasound of the second trimester [83]. Izmirly performed a 10-year follow-up of live-born children with congenital total atrioventricular block. He estimated their 10-year survival at 86%, with 70% of them requiring pacemaker implantation [87]. 

### 2.20. Endocardial Fibroelastosis

Another cardiological complication described in the children of mothers with anti-SS-A positive is endocardial fibroelastosis, the dangerous consequence of which is dilated cardiomyopathy. Endocardial fibroelastosis (EFE), previously only diagnosed post-mortem in histopathological examinations [8], is now visible in fetal heart echocardiography [88]. Guttrot-Imbret and her colleagues examined five cases of endocardial fibroelastosis in children; four were prenatally diagnosed, one after birth. In all five cases described, anti-SS-A and anti-SS-B antibodies were detected in the mother. In three cases, the diagnosis in the child was the reason for the assessment of the mother’s serological status. The course of the disease was mild in the described children. The author emphasized that the disease is insidious, and, if not noticed in echocardiograms, it can quickly lead to significant heart failure caused by dilated cardiomyopathy. In her work, Guttrot-Imbret quoted the results of her previous work on endocardial fibroelasis and referred to the results of Nield et al. from 2002. Nield described 13 cases of children with complete atrioventricular block associated with fibroelastosis of the endocardium, mainly affecting the left ventricle. Six mothers had positive titers of anti-SS-A and anti-SS-B antibodies, and seven mothers had only anti-SS-B antibodies. Endocarditis fibroelastosis developed from 6 to 12 weeks after prenatal diagnosis of CHB and from 7 months to 5 years after birth. Severe left ventricular dysfunction was found in all cases; in nine cases, it resulted in death, and in two cases in heart transplantation. Histopathological examination was performed in 10 patients, severe fibroelastosis was found in 7 cases, and mild in the remaining 3. Immunohistochemical examination was performed in three fetuses. IgG, IgM, and T lymphocyte infiltration were revealed, which suggested an immune response of the fetus or child [88]. Subsequently, the same author published another paper describing three cases of severe endocardial fibroelastosis without atrioventricular block. Children affected by fibroelastosis were born by mothers with anti-SS-A antibodies; two of them died, and one of them underwent heart transplantation with good results [73]. Nield noted that atrioventricular block is not the cause of fibroelastosis, and these two diseases should be treated as independent manifestations of neonatal lupus. Atrioventricular block is the result of the destruction of atrioventricular node tissues, whereas in fibroelastosis, the degenerative lesions are disseminated. Mortality in fibroelastosis is high in both fetuses and neonates; however, fetuses show a more rapid disease development, which is often complicated by edema. In fetal fibroelastosis, both ventricles are often affected, whereas, when a neonate suffers from this condition, it seems to have existed in the fetus in latent form, and its development requires an additional factor such as a viral antigen (cytomegalovirus enhancing the expression of the Ro antigen on cells) or another autoantibody. Endocarditis fibroelastosis coexists with atrioventricular block in 5% of cases; however, this coexistence is extremely important because fibroelastosis is a risk factor of death in the population of children with congenital heart block. Patients may develop fibroelastosis even after the pacemaker has been properly implanted, so it is important to monitor patients by performing electrocardiograms. Regular testing would help to better identify this group of patients by allowing strategic treatment or, in severe cases, early registration on a waiting list for transplantation. In his studies, Nield noted that in the prenatal period there is an overdiagnosis of fibroelastosis in echocardiography, whereas, in the neonatal period, this diagnosis is significantly underestimated [89,90]. Fibroelastosis caused by circulating autoantibodies due to high mortality rates requires an attempt at immunosuppressive therapy. Prospective studies are required to set the standard of treatment and evaluate its efficacy. In summary, EFE induced by the mothers’ autoantibodies may occur in the presence of CHB and is associated with high mortality rates among affected fetuses and infants. The immune response of a fetus or infant to autoantibody deposition in the myocardium is likely to contribute to the development of the disease. Additional studies are necessary to determine the true prevalence of EFE in fetuses and infants born to mothers positive for anti-Ro or anti-La-positive antibodies and to establish optimal therapeutic options in these patients.

### 2.21. Dilated Cardiomyopathy

Dilated cardiomyopathy is the most common cardiomyopathy in children. Most of them suffer from idiopathic cardiomyopathy, but in some cases, endocardial and mediastinal biopsy reveals the features of fibroelastosis of the endocardium. Matitiau et al. evaluated 24 children under 2 years of age; 50% recovered without complications; 30% died; and 20% survived, but with significant left ventricular dysfunction remaining. In 16 children, heart biopsies were performed; in 45%, myocarditis was diagnosed, and in 25% of cases, endocarditis was diagnosed. In 16 children, a diagnosis of inflammation or fibroelastosis was confirmed by histopathological examination. Patients with inflammatory changes in biopsy had better prognosis than those with histological features of fibroelastosis. Unfortunately, Matitiau did not refer to the serological status of the mothers in his studies. The only known factor is that in the families of these children, there were no previously diagnosed inborn cardiomyopathies [91]. 

### 2.22. Fetal Growth Restriction (FGR)

Wisuthsarewong et al. documented that FGR was the accompanying symptom in 29.4% of cases of neonatal lupus [60]. Vasculopathy in the mother’s vessels, including maternal vascular thrombosis, is also associated with FGR. Several authors have found that maternal vasculopathy correlates with gestational induced hypertension, but the relationship between maternal vasculopathy and collagen disease is unclear [92]. 

### 2.23. Disease Registries 

Gathering data in registries allow the characterization of the clinical picture of mothers and children. Such registries serving scientific purposes have already been established in several countries. The first lupus registry was created in the United States in 1994 to collect data of mothers with positive anti-SS-A and/or anti-SS-B and/or anti-RNP antibodies and whose children had lupus symptoms [85]. Fredi et al. published the results of the Italian neonatal lupus registry in February 2019 [93]. The registry collected data from 1969 to 2017 on women with anti-SS-A and/or anti-SS-B antibodies and on children with congenital first- or second-degree heart block. The French Registry of Neonatal Lupus (French RNL) has been kept since 2000. In addition, there are reviews developed by scientists from Canada, Spain, and Sweden. A research limitation in this case is the low incidence of neonatal lupus. It appears great emphasis should be put on multi-center cooperation and data harmonization.

### 2.24. Pregnancy Monitoring 

Current recommendations include performing fetal echocardiography weekly between 16 and 26 weeks of pregnancy and every 2 weeks between 26 and 34 weeks of pregnancy in pregnant women with systemic lupus erythematosus [42,94]. Atrioventricular block can develop in less than 24 h. Cuneo et al. evaluated the effectiveness of home monitoring of fetal heart rate. A total of 273 pregnant women from 16 international centers completed the whole study protocol. They all tested positive for anti-SS-A antibodies. Mothers checked the fetal heart rate twice a day, and echocardiography was performed weekly or every 2 weeks. In the case of abnormal fetal heart rate, echocardiography was performed as quickly as possible. A total of 21 mothers registered abnormal fetal rhythms; 14 fetuses had mild arrhythmias; 4 of them had a first-degree atrioventricular block; 1 had a second-degree atrioventricular block; and 2 had a third-degree atrioventricular block. None of the fetuses with first-degree atrioventricular block developed a more advanced block, and there was no progression of rhythm disturbances. One child with a first-degree atrioventricular block had endocardial fibroelastosis, and for this reason, they were administered dexamethasone and stayed at home for observation. The case of a child with a second-degree atrioventricular block was particularly interesting. Treatment was administered within 12 h after the abnormal rhythm had been detected (dexamethasone and immunoglobulins administered on the day the abnormal rhythm occurred). As a result, the sinus rhythm was restored. The fetus was also found to suffer from other conditions, including endocardial fibroelastosis and tricuspid valve insufficiency. Despite treatment with dexamethasone and immunoglobulins, the sinus rhythm could not be restored in fetuses with third-degree atrioventricular block. Both of them were diagnosed with endocardial fibroelastosis; their mothers had a very high anti-SS-A antibodies titer, and in one of them, systemic lupus erythematosus was diagnosed and Sjögren’s syndrome in the other; both has been receiving hydroxychloroquine since the 12th week of pregnancy, and their children required stimulator implantation at birth [95]. 

Another aspect was investigated by Evers et al. in a study published this year. He tried to identify a strategy that would optimize the use of echocardiography since he considered weekly testing of all patients with anti-SS-A antibodies to be an exaggeration. Decision analysis of cost/utility modelling for three screening paradigms was performed: ‘standard screening’ (SS) where mothers in mid-pregnancy were subjected to weekly screening tests; ‘limited screening’ (LS) where fetal echocardiograms were avoided unless the fetus develops bradycardia, and ‘targeted screening based on maternal antibody titers’ (TS), where only high anti-Ro values justified weekly screening. While the effectiveness of fetal intervention for first- or second-degree AV block remains unclear, this analysis supports the use of antibody levels to stratify this population in order to optimize surveillance of the occurrence of a potential atrioventricular block. Standard weekly screening tests are inefficient in terms of costs and result in over-exploitation of financial resources [96]. 

It appears that monitoring the fetal heart rate by the mother at home is the most effective approach. It is important to find specific markers to identify high-risk pregnancies and to ensure that women are able to monitor the fetal heart rate and have prompt access to obstetric care in the event of abnormal readings.

### 2.25. Treatment

Since atrioventricular block is usually diagnosed between 18 and 24 weeks of pregnancy, effective therapy should already be administered in the prenatal period. Mortality due to this disease varies between 15–20% [87]. Negative prognostic factors include: low gestation age of the child (<20 week) at the time of the onset of symptoms, generalized fetal edema, cardiomyopathy, fibroelastosis, low heart rate ≤ 50 bpm, impaired left ventricular function, and prematurity [87,97]. Various forms of treatment are considered, including steroid therapy, plasmapheresis, and intravenous immunoglobulins.

In 2008, Brucato summarized the current state of knowledge about the treatments. Non-fluorinated steroids (prednisone, prednisone, and methylprednisolone) are recommended only if the health condition of the mother requires it and should not be administered to prevent fetal CHB in women who tested positive for anti-Ro. Fluorinated steroids (dexamethasone or betamethasone) are not metabolized by the placenta and are available to the fetus in their active form. Routine prophylactic fluorinated steroid therapy is not recommended even for women who have previously had children with CHB or a skin rash because this therapy has side effects, mainly regarding brain development. Intravenous immunoglobulin is not used prophylactically, but if administered early enough after the detection of atrioventricular block, it increases the chances of restoring the sinus rhythm. The current recommendation is that, if the mother tests positive for anti-Ro antibodies, serial echocardiograms and sonograms should be performed in accordance with the algorithm described above. The aim is to detect early fetal abnormalities, such as premature atrial contractions or moderate pericardial effusion, which may precede a total atrioventricular block and may be the aim of the preventive therapy. If there are no disease symptoms, fluorinated steroids should not be used. In the event of disturbing symptoms, betamethasone seems safer than dexamethasone [98].

Studies conducted by Sonesson et al. on a group of 212 patients have shown that fluorinated steroids may reverse first- and second-degree atrioventricular block, but also that third-degree atrioventricular block may prove to be reversible if treatment begins shortly after its onset [99]. These data were also confirmed by Cunelo [95]. However, the results of a multi-center study supervised by Eliasson oppose this thesis. They do not support the therapeutic strategy to administer steroid treatment to fetuses with third-degree CHB [97]. Trucco also analyzed the data of patients treated with glucocorticosteroids (GCS) and intravenous immunoglobulins (IVIG) (the group consisted of 20 fetuses). It seems that treatment of fetal cardiomyopathy and/or endocardial fibroelastosis with CTS and IVIG potentially improves fetal prognosis [100].

### 2.26. The PRIDE Study 

In 2008, Friedman et al. published the results of a large study evaluating the correlation between the length of the PR interval and the use of dexamethasone. In the literature on the subject, this study is known under the acronym PRIDE (The PR Interval and Dexamethasone Evaluation Prospective Study). The study group consisted of 127 mothers from 33 centers who tested positive for anti-SS-A antibodies before or up to 18 weeks of pregnancy and who were treated with prednisone at a dose below 10 mg/day or did not receive glucocorticosteroids at all [94]. PR intervals of >150 ms were considered as first-degree atrioventricular block. A total of 92 fetuses had normal PR intervals. Neonatal lupus developed in 10 cases out of 98 fetuses. The distribution of symptoms was as follows: four children had only skin lesions; three fetuses had third-degree atrioventricular block; and three fetuses had first-degree atrioventricular block. In the case of one patient, the tricuspid regurgitation preceded the occurrence of the third-degree atrioventricular block. Two fetuses with the first-degree block were administered dexamethasone therapy at a dose of 4 mg/day, which resulted in heart rate normalization. In summary, the heart block occurred in 3 out of 16 pregnancies (19%) in mothers whose previous child had a congenital heart block, and in 3 out of 74 pregnancies (4%) in mothers without a previous child with congenital heart block or rash. The risk that a fetus would develop a heart block was higher in mothers whose previous offspring were affected by congenital heart block. Extensions of the PR interval were rare and did not precede a more advance block.

High-grade AV block and cardiomyopathy may occur within one week of a normal echocardiogram, without an initial first-degree block. It has been demonstrated that early intrauterine treatment of an incomplete AV block with fluorinated steroids prevents progression of cardiac block [95,99,101].

Since the PRIDE study, we have acquired better insight into early prenatal lupus development in neonates with cardiac conditions. It has been determined that the phenotype of the cardiac form of NLE varies from the clinically silent first-degree atrioventricular block, which does not progress, to the third-degree atrioventricular block, which develops into generalized fetal edema (hydrops) and results in death of the fetus within less than one week after the appearance of the first symptoms [102]. It is also now known that the fetal AV value and PR interval in the newborn are variable and that the AV interval, which is considered to indicate first-degree CT block, does not need to develop into the second- or third-degree atrioventricular block even with no accompanying treatment. Interestingly, a prolonged AV interval in humans may represent another pathogenesis involving anti-SS-A antibodies, or, in some cases, there may be a yet unknown protective genetic or environmental factor that inhibits the destruction of the atrioventricular node.

### 2.27. Hydroxychloroquine

Hydroxychloroquine (HCQ) passes through the placental barrier and is considered safe for pregnant women and fetuses. It inhibits Toll-like receptors (TLR).

Hydroxychloroquine has a long half-life, and therefore it takes longer for it to achieve efficacy. Therefore, hydroxychloroquine should be implemented in the pre-conception period 2–8 weeks before the planned pregnancy.

The incidence of neonatal lupus was lower in women taking hydroxychloroquine, even if the disease was asymptomatic [103]. At present, hydroxychloroquine seems to be the gold standard in the prevention of fetal lupus complications [82]. Previously, hydroxychloroquine was believed to reduce the incidence of cardiological complications, but not other symphtoms. Barsalou’s study published in 2018 showed that the use of hydroxychloroquine also reduces the risk of skin lesions. The prevention of skin lesions is important to reduce the occurrence of permanent scars, telangiectasias, epidermal atrophy, and pigmentation of the skin [82].

### 2.28. Ritodrine

Ritodrine, a β-sympathomimetic drug in Japan commonly used as a tocolytic administered to mothers, may cause acceleration in fetal heart rate patterns. Some studies were conduct in Japan and indicate the advantages of using ritodrine. Matsubara et al. indicated that maternal administration of ritodrine increased the ventricular rate and thus fameliorated the signs of fetal heart failure if this developed due to CHB [104,105].

Miyoshi et al. also confirmed that bradycardia was improved by beta-sympathomimetic administration, but the survival rate was not improved. The second statement was that chronic use of steroids for more than 10 weeks can lead to adverse effects in the fetus such as fetal growth restriction and oligohydramnios [106].

In Europe, according to the latest recommendation, ritodrine is not a first choice medication due to adverse events. It can cause complications such as necrotizing enterocolitis, intraventricular hemorrhage, and acute respiratory distress syndrome in fetus. The adverse maternal cardiopulmonary effects described with beta-agonists frequently cause treatment to be interrupted, in conclusion that beta-agonists no longer be prescribed for tocolysis [107,108].

Before decision about the usage of ritodrine, a balance of benefits and risks should be made.

## 3. The Future of Treatment

### 3.1. Interleukin Inhibitors

The most recent recommendations from 2018 concerning systemic lupus erythematosus treatment emphasize the role of ustekinumab (IL-12 and IL-23 inhibitor) [109]. There are no clear recommendations for the use of ustekinumab during pregnancy, but there are several reports that indicate the safety of this treatment. These data refer to patients with psoriasis (four cases) [110] and Crohn’s disease (one case described) [111]. In both reports, all the patients used ustekinumab before and during pregnancy and gave birth to healthy children. No toxicity to the mother of fetus was observed. However, nowadays, treatment involving biological drugs is not recommended during pregnancy. Nevertheless, such a therapy should be considered in some women with severe types of autoimmune inflammatory diseases. Taking into consideration the disturbances of immune processes in the Il-23 axis in the pathogenesis of congenital total cardiac block formation, treatment with an Il-23 inhibitor might be a chance to protect the fetus against such complications.

### 3.2. Will IVIG Always Be Necessary?

Currently approved treatment methods aimed at removing pathogenic autoantibodies in autoimmune diseases include plasmapheresis and IVIG (intravenous infusions of immunoglobulin G). They are expensive, require complicated procedures, and are burdened with the risk of adverse effects, which is why attempts are being made to develop alternative therapeutic strategies. The results of studies conducted in the USA [112] have revealed that anti-FcRn therapies may prove useful in the treatment of diseases involving pathogen IgG autoantibodies. It was demonstrated that the effectiveness of IVIG in the treatment of autoimmune diseases associated with the presence of pathogenic IgG antibodies is fully dependent on the neonatal Fc receptor (FcRn). When FcRn was blocked with high doses of IVIG, pathogenic autoantibodies were not protected by the FcRn receptor against catabolism and thus degraded more rapidly. Therefore, blocking the FcRn-IgG interaction is a rational approach aimed at reducing the level of pathogenic autoantibodies in autoimmune diseases. The FcRn–IgG interaction can be blocked using FcRn-specific monoclonal antibodies, IgG (developed by genetic engineering) with high affinity to FcRn both in acidic and physiological environments, FcRn receptor peptide blockers, or by regulating FcRn expression [43]. Whether we will find an alternative to IVIG therapy depends on the results of the multifaceted research currently being conducted.

## 4. Summary

Neonatal lupus still poses many unanswered questions about its pathogenesis. Anti-Ro’s relationship with macrophage activation and IFN secretion should be further investigated. The consequences of neonatal lupus may be grave. Ill neonates should be treated in specialist tertiary medical centers. Interdisciplinary care is also advisable. 

Thus far, there are no clear care standards for pregnant women or mothers and children after childbirth. The published manuscripts focused on small populations in various countries and provided only limited information on different populations. Registers of neonatal lupus cases may be helpful in determining uniform international recommendations for action.

The usefulness of the close monitoring of fetal heart rate in the critical weeks of pregnancy in terms of the highest exposition to the development of neonatal lupus symptoms, as well as the protective effect of hydroxychloroquine, are commonly indicated in the literature on this subject. There is a need to observe larger groups of patients in order to identify high-risk populations, improve screening protocols, and establish uniform treatment.

## Figures and Tables

**Table 1:** Difference between anti-Ro52 and anti-Ro60.

	Anti-Ro52	Anti-Ro60
Most likely rheumatic disease relevant to specific antibodies	Sjögren’s syndrome (SS)	
	Systemic lupus erythematosus (SLE) especially cutaneous involvement	Systemic lupus erythematosus (SLE)
	Systemic sclerosis (SSC)	Neonatal lupus erythematosus (NLE)
	Diffuse cutaneous systemic sclerosis	
	Primary biliary cirrhosis (PBC)	
	Polymyositis/dermatomyositis (PM/DM)	
	Interstitial lung disease (ILD)	
	Congenital complete heart block (CHB)	
Other conditions relevant to antibodies	Malignancies	
	Infections	
Function of protein against which the antibody is directed	E3 ubiquitin ligases	RNA binding
Gene coding for the protein against which the antibodies are directed	TRIM21	TROVE2

**Table 2:** Clinical picture of SS-A-positive mothers who gave birth to children with symptoms of neonatal lupus (number of patients/percentage).

Author	Brucato et al. 2001 [70]	Cimaz et al. 2003 [71]	Martinez-Sanchez et al. 2017 [72]	Zuppa et al. 2017 [69]	Li et al. 2015 [73]	Luo et al. 2015 [74]
Number of patients	100	112	40	50	123	140
Systemic lupus erythematosus (SLE)	53 (53%)	74 (66.1%)	16 (40%)	25 (50%)	34 (27.64%)	93 (66.44%)
Sjögrenś Syndrome (SS)	25 (25%)	10 (8.9%)	15 (37.5%)	15 (30%)	2 (1.63%)	30 (21.43%)
Comorbidity of SLE and SS	6 (6%)	0	0	5 (10%)	0	0
Mixed connective tissue disease	1 (1%)	0	0	2 (4%)	0	2 (1.43%)
Antiphospholipid syndrome	1 (1%)	0	0	0	0	14 (10%)
Rheumatoid arthritis	0	0	1 (2.5%)	0	1 (0.81%)	0
Scleroderma	1 (1%)	0	0	0	0	0
Undifferentiated connective tissue disease	19 (19%)	5 (4.5%)	0	0	7 (5.69%)	11 (7.86%)
Healthy	0	15 (13.4%)	8 (20%)	3 (6%)	78 (63.41%)	4 (2.86%)
Psoriasis	0	0	0	0	1 (0.81%)	0

**Table 3:** Prevalence of neonatal lupus symptoms.

Prevalence of NLS Features Review of the Literature								
			NLS Features (% of Study Group)					
Authors	Patients No	Follow Up	CCHB	Other Cardiac	Skin	Hematological	Hepatobiliary	CNS
Cimaz Ret al. (2003) [71]	128	6–9 months	1.6	41	16	27	25	NR
Motta M et al. (2007) [75]	51	Not performed	2	15.7	3,9	3.9	0	NR
Boros C et al. (2007) [76]	47	12 months	25.5		40	36	36	8
Zuppa (2017) [69]	50	9 months	4	12	0	24	56	18

Definitions: NR: not reported; CCHB: complete congenital heart block; “Other Cardiac” evaluated: I or II degree AV block or prolonged QT interval; CNS: central nervous system.

## Abbreviations

NLE	neonatal lupus erythematosus
Anti-SS-A	anti-Sjögren Syndrome A antibodies
Anti-SS-B	anti-Sjögren Syndrome B antibodies
Anti-RNP	anti-ribonucleoprotein antibodies
LE cells	lupus erythematosus cell
SLE	systemic lupus erythematosus
ACR	American College of Rheumatology
ANA	anti-nuclear antibodies
ELISA	enzyme-linked immunosorbent assay
Anti-dsDNA	double-stranded native DNA antibodies
EFE	endocardial fibroelastosis
IRF	interferon regulatory factor
CHB	congenital heart block
IIF	indirect immunofluorescence
EBNA1	Epstein-Barr virus nuclear antigen-1
MHC	major histocompatibility complex
AV	Atrioventricular
TNFα	tumor necrosis factor alpha
TGFβ	transforming growth factor beta
IFN	Interferon
SIGLEC-1	sialic acid-binding Ig-like lecithin 1
